# Unusual Derivatization of Methylmalonic Acid with Pentafluorobenzyl Bromide to a Tripentafluorobenzyl Derivative and Its Stable-Isotope Dilution GC-MS Measurement in Human Urine

**DOI:** 10.3390/molecules27165202

**Published:** 2022-08-15

**Authors:** Alexander Bollenbach, Svetlana Baskal, Catharina Mels, Ruan Kruger, Dimitrios Tsikas

**Affiliations:** 1 Core Unit Proteomics, Institute of Toxicology, Hannover Medical School, 30625 Hannover, Germany; 2 Hypertension in Africa Research Team (HART), North-West University, Potchefstroom 2531, South Africa; 3 MRC Research Unit for Hypertension and Cardiovascular Disease, North-West University, Potchefstroom 2531, South Africa

**Keywords:** alkylation, base, C-H acidity, dicarboxylic acids, ethnicity, esterification, GC-MS, pentafluorobenzyl bromide, stability, toluene, urine

## Abstract

Methylmalonic acid (MMA) is a very short dicarboxylic acid (methylpropanedioic acid; CH_3_CH(COOH)_2_; *p*K_a1_, 3.07; *p*K_a2_, 5.76) associated with vitamin B_12_ deficiency and many other patho-physiological conditions. In this work, we investigated several carboxylic groups-specific derivatization reactions and tested their utility for the quantitative analysis of MMA in human urine and plasma by gas chromatography-mass spectrometry (GC-MS). The most useful derivatization procedure was the reaction of unlabeled MMA (d_0_-MMA) and trideutero-methyl malonic acid (d_3_-MMA) with 2,3,4,5,6-pentafluorobenzyl bromide (PFB-Br) in acetone. By heating at 80 °C for 60 min, we observed the formation of the dipentafluorobenzyl (PFB) ester of MMA (CH_3_CH(COOPFB)_2_). In the presence of *N,N*-diisopropylamine, heating at 80 °C for 60 min resulted in the formation of a tripentafluorobenzyl derivative of MMA, i.e., CH_3_CPFB(COOPFB)_2_). The retention time was 5.6 min for CH_3_CH(COOPFB)_2_ and 7.3 min for CH_3_CPFB(COOPFB)_2_). The most intense ions in the negative-ion chemical ionization (NICI) GC-MS spectra of CH_3_CH(COOPFB)_2_ were mass-to-charge (*m/z*) 233 for d_0_-MMA and *m/z* 236 for d_3_-MMA. The most intense ions in the NICI GC-MS spectra of CH_3_CPFB(COOPFB)_2_ were mass-to-charge (*m/z*) 349 for d_0_-MMA and *m/z* 352 for d_3_-MMA. These results indicate that the H at C atom at position 2 is C-H acidic and is alkylated by PFB-Br only in the presence of the base *N,N*-diisopropylamine. Method validation and quantitative analyses in human urine and plasma were performed by selected ion monitoring (SIM) of *m/z* 349 for d_0_-MMA and *m/z* 352 for the internal standard d_3_-MMA in the NICI mode. We used the method to measure the urinary excretion rates of MMA in healthy black (*n* = 39) and white (*n* = 41) boys of the Arterial Stiffness in Offspring Study (ASOS). The creatinine-corrected excretion rates of MMA were 1.50 [0.85–2.52] µmol/mmol in the black boys and 1.34 [1.02–2.18] µmol/mmol in the white boys (*P* = 0.85; Mann–Whitney). The derivatization procedure is highly specific and sensitive for MMA and allows its accurate and precise measurement in 10-µl of human urine by GC-MS.

## 1. Introduction

**Scheme 1:**
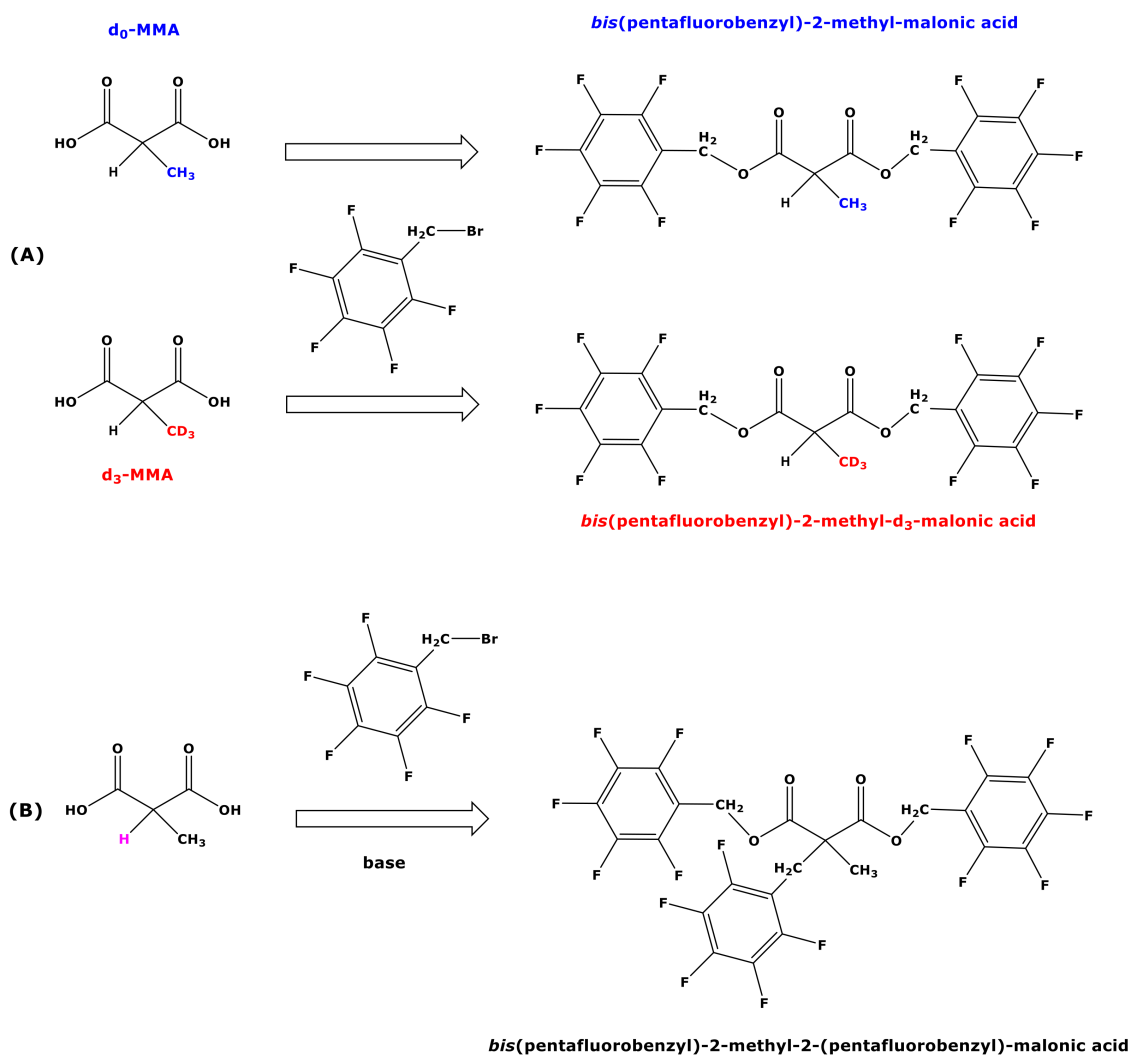
One-step derivatization of methyl malonic acid (MMA) with 2,3,4,5,6-pentafluorobenzyl bromide (**A**) to its dipentafluorobenzyl ester and (**B**) to its dipentafluorobenzyl ester-pentafluorobenzyl derivative. d_0_-MMA, unlabeled MMA; d_3_-MMA, trideutero-MMA.

Pentafluorobenzyl bromide (PFB-Br) is a versatile derivatization reagent in chromatography and mass spectrometry for various organic and inorganic classes of analytes [1,2]. Derivatization reactions with PFB-Br can be performed in water-free organic solvents such as acetonitrile or in the aqueous phase. Mid- and long-chain mono- and dicarboxylic acids including fatty acids, prostaglandins, leukotrienes, thromboxane, and metabolites are commonly derivatized with PFB-Br to their mono- and di-PFB esters in anhydrous acetonitrile under mild conditions (e.g., heating at 30 °C for 60 min) using an organic base such as *N,N*-diisopropylamine as the catalyst [2]. Interestingly, PFB-Br is useful for the alkylation of inorganic anions such as nitrate [3] and carbonate [4], as well as for C-H acidic compounds such as malondialdehyde (MDA; malonic aldehyde, *p*K_a_, 4.5) [5]). Methyl malonic acid (MMA), that is, methyl propanedioic acid (CH_3_CH(COOH)_2_; *p*K_a1_, 3.07; *p*K_a2_, 5.76) is a very short dicarboxylic acid. Yet, to the best of our knowledge, there is no report on a C-H-acidity of MMA. Given the considerable biological importance of MMA in plasma and urine, for instance, in association with vitamin B_12_ deficiency, we were interested to know, whether MMA can be alkylated with PFB-Br analogous to MDA. PFB-Br was shown to react with MMA and to form a di-PFB ester for use in liquid chromatography-tandem mass spectrometry (LC-MS/MS) [6]. In the present study, we investigated the derivatization of unlabeled MMA (d_0_-MMA) and trideutero-methyl malonic acid (d_3_-MMA) with 2,3,4,5,6-pentafluorobenzyl bromide (PFB-Br) under various experimental conditions (Scheme 1). We provide evidence for the formation of a tripentafluorobenzyl derivative of MMA, that is, CH_3_CPFB(COOPFB)_2_, only in the presence of an organic base and at a higher temperature, strongly indicating that MMA is a C-H-acidic compound, yet less strong C-H-acidic than MDA. Based on this one-step derivatization reaction, we developed and validated a stable-isotope dilution GC-MS method for the quantitative measurement of MMA in human urine.

In the present work, we used the newly developed GC-MS method to measure the excretion rate of MMA in the bi-ethnic epidemiologic study ASOS, the Arterial Stiffness in Offspring study. Originally, the ASOS study was conducted to investigate the link of urinary metabolites with premature arterial stiffness and the early detection and identification of cardiovascular disease and hypertension development in black and white populations from South Africa [7]. Previous results from the ASOS study revealed ethnic-dependent differences in healthy black and white boys with respect to native and modified amino acids, nitrite, and nitrate [7,8,9,10].

## 2. Materials and Methods

### 2.1. Chemicals and Materials 

2,3,4,5,6-Pentafluorobenzyl bromide (PFB-Br) was obtained from Sigma-Aldrich (Steinheim, Germany). Toluene was purchased from Baker (Deventer, The Netherlands). Acetone, Hünig base, d_0_-MMA, and d_3_-MMA (isotopic purity, 98% ^2^H) were supplied by Merck (Darmstadt, Germany). Stock solutions of d_0_-MMA and d_3_-MMA were prepared by dissolving accurately weighed amounts of the unlabeled and isotope-labeled MMA in deionized water. Stock solutions were diluted with deionized water as appropriate. Glassware for GC-MS (1.5-mL autosampler vials and 0.2-mL microvials) including the fused-silica capillary column Optima 17 (15 m × 0.25 mm I.D., 0.25 µm film thickness) were purchased from Macherey-Nagel (Düren, Germany). 

### 2.2. Derivatization Procedures for Aqueous d_0_-MMA and d_3_-MMA

The derivatization procedures are described in detail in the Results section. PFB-Br is corrosive and an eye irritant. Inhalation and contact with skin and eyes should be avoided. All work should be and was performed in a well-ventilated fume hood.

### 2.3. Derivatization Procedure for MMA in Human Urine Samples

Native urine samples (10 µL, no pH adjustment) were pipetted into 1.5 mL glass vials (Macherey-Nagel; Düren, Germany). Aliquots (10 µL) of 100 µM d_3_-MMA were added to reach a final added concentration of 100 µM with respect to the urine sample. After the addition of 10 µL Hünig base, 100 µL acetone, and 10 µL PFB-Br (diluted 1:3, *v*/*v*, in acetonitrile) glass vials were sealed tightly, and the samples were incubated for 60 min at 80 °C. After cooling to room temperature, solvents and reagents were removed under a stream of nitrogen. Subsequently, the solid residues were treated with 200 µL of toluene, followed by vortex-mixing for 1 min and centrifugation (4000× *g*, 5 min, 18 °C). Aliquots (150 µL) of the upper organic phase were transferred into autosampler glass vials equipped with microinserts, the samples were sealed and subjected to GC-MS analysis. The concentration of the internal standard was within expected concentrations of endogenous urinary MMA.

### 2.4. Quantitative GC-MS Analyses of MMA

A GC-MS apparatus consisting of a single quadrupole mass spectrometer model ISQ, a Trace 1210 series gas chromatograph, and an AS1310 autosampler from ThermoFisher (Dreieich, Germany) equipped with a 10-µL Hamilton needle was used. Toluene aliquots (1 µL) were injected in the splitless mode. The injector temperature was kept at 280 °C. Interface and ion-source temperatures were set to 300 °C and 250 °C, respectively. Helium was used as the carrier gas at a constant flow rate of 1.0 mL/min. Methane was used as the reagent gas for negative-ion chemical ionization (NICI) at a constant flow rate of 2.4 mL/min. Electron energy was 70 eV and electron current 50 µA. Two oven temperature programs were used. Program #1: the oven was held at 40 °C for 0.5 min and ramped to 210 °C at a rate of 15 °C/min and then to 320 °C at a rate of 35 °C/min. Program #2: the oven was held at 90 °C for 0.5 min and ramped to 210 °C at a rate of 30 °C/min and then to 320 °C at a rate of 15 °C/min. The final temperature was held for 0.5 min. In quantitative analyses, oven program #2 was used. The dwell time was 100 ms for each ion in the selected-ion monitoring (SIM) mode and the electron multiplier voltage was set to 1400 V.

The concentration of MMA in the human urine and plasma samples in quantitative analyses was determined in the SIM mode using one mass fragment (*m/z*) for d_0_-MMA and one ion for the corresponding mass fragment for d_3_-MMA serving as the internal standard (IS). The peak area (PA) values of d_0_-MMA and d_3_-MMA were calculated automatically by the GC-MS software (Xcalibur and Quan Browser). The concentration of MMA was calculated by multiplying the peak area ratio (PAR) of the endogenous d_0_-MMA and the internal standard d_3_-MMA with the known concentration of d_3_-MMA. Statistical analyses and graphs were performed and prepared by GraphPad Prism 7 (San Diego, CA, USA). Chemical structures were drawn by using ChemDrawProfessional 15.0 (Perkin Elmer).

### 2.5. Subjects—The Arterial Stiffness in Offspring Study (ASOS)

We applied the current method to quantify MMA in 10 µL aliquots of spot urine samples of 39 healthy black boys and 41 healthy white boys (aged 6–8 years) collected in a previous study after approval by the local Ethics Committee [11]. Ethical statement: participants were fully informed about the objective of the study (written informed consent and assent were obtained from all participants included in the study). All procedures performed in the study were in accordance with the ethical standards of the institutional and/or national research committee (Health Research Ethics Committee of the North-West University; NWU-00007-15-A1) and with the 1964 Helsinki Declaration and its later amendments or comparable ethical standards [12].

Creatinine-corrected excretion rates are reported as µmol of MMA per mmol of creatinine.

## 3. Results and Discussion

### 3.1. Derivatization of d_0_-MMA and d_3_-MMA and GC-MS Characterization of Their Derivatives 

Mass spectra of MMA were obtained after derivatization with PFB-Br of separate aliquots containing each 5 nmol of aqueous solutions of d_0_-MMA and d_3_-MMA. After evaporation to dryness under a stream of nitrogen gas, the residues were reconstituted in 100 µL acetone and 10 µL pure PFB-Br were added. Half of the samples were treated each with a 10 µL Hünig base. The samples were sealed tightly and incubated for 60 min at 60 °C or 80 °C. After cooling to room temperature, solvents and reagents were removed under a stream of nitrogen and the solid remaining were treated with 200 µL toluene. The samples were sealed, vortexed for 1 min, and centrifuged (4000× *g*, 5 min, 18 °C). Aliquots of 150 µL of the toluene phases were transferred into autosampler vials equipped with microinserts, and 1-µL aliquots were injected into the GC-MS apparatus in the splitless mode. NICI mass spectra of the d_0_-MMA and d_3_-MMA derivatives were generated by scanning the quadrupole in the *m/z* range 50–1000 with a scan rate of 1 s per cycle. The structures of the d_0_-MMA and d_3_-MMA derivatives were elucidated considering the expected 3-Da difference between unlabeled (d_0_-MMA) and deuterium-labeled (d_3_-MMA) analytes and the expected shorter retention times of deuterium atoms containing analytes [13,14].

**Figure 1:**
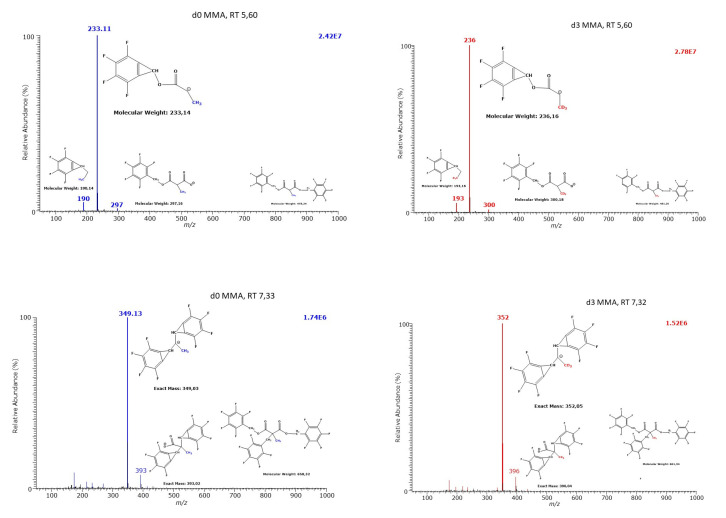
GC-MS spectra of the PFB derivatives of d_0_-MMA and d_3_-MMA formed from pentafluorobenzyl bromide in the presence of Hünig base. Insertions indicate the proposed structures of the derivatives and anions formed by NICI.

Derivatization of d_0_-MMA and d_3_-MMA with PFB-Br in the presence of Hünig base by heating at 60 °C for 60 min resulted in the formation of two GC-MS peaks with retention times of 12.26 min and 13.66 min for d_0_-MMA and 12.24 min and 13.65 min for d_3_-MMA, using the oven program #1. The earlier eluting peaks were about 10 times larger than the later eluting peaks. The most intense mass fragments of the larger peak were *m/z* 233 for d_0_-MMA and *m/z* 236 for d_3_-MMA. The most intense mass fragments of the minor peak were *m/z* 349 for d_0_-MMA and *m/z* 352 for d_3_-MMA. Using oven program #2 we also observed two GC-MS peaks with retention times of 5.60 min and 7.33 min for d_0_-MMA, and 5.60 min and 7.32 min for d_3_-MMA (Figure 1). The earlier eluting GC-MS peaks were also larger than the later eluting GC-MS peaks. The difference of 3 Da indicates the presence of the methyl groups in both derivatives. The difference in the retention times of the derivatives was smaller using oven program #2. In the absence of the Hünig base, only the earlier eluting derivatives were formed (data not shown).

Mono- and dicarboxylic acids react with PFB-Br in anhydrous acetonitrile and in the presence of a base such as the Hünig base to form mono- and di-PFB esters [2]. Under NICI conditions, mono- and di-PFB esters of carboxylic acids ionize to form their very stable carboxylate anions by losing the PFB moieties as radicals [2]. Unlabeled and deuterium labeled α-methyl groups such as that of ibuprofen (isobutylphenylpropionic acid) are stable under NICI [13]. The di-PFB esters of d_0_-MMA and d_3_-MMA would have molecular weights of 478.2 and 481.2 (Figure 1). In the NICI mode, neutral loss of a PFB radical (181 Da) would generate carboxylate anions [M−PFB]^−^ with *m/z* 297 and *m/z* 300. These mass fragments are found in the GC-MS mass spectra of the earlier eluting peaks, yet they are very weak. The most intense mass fragments at *m/z* 233 and *m/z* 236 suggest that the carboxylate anions undergo further fragmentation by losing two leaving groups, i.e., CO_2_ (44 Da) and HF (20 Da), that is, 64 Da in total. The weak mass fragments at *m/z* 190 and *m/z* 193 suggest additional loss of CO_2_ (44 Da) from the second carboxylic group. The most likely structures of the earlier eluting PFB derivatives of d_0_-MMA and d_3_-MMA are 1,3-diPFB esters. The α-methyl groups of d_0_-MMA and d_3_-MMA seem to be very stable under the derivatization and NICI conditions. The formation of a 1,3-diPFB ester of MMA has been reported and used for the determination of MMA in serum by LC-MS/MS [6]. These authors found that succinic acid (SA, 1,4-butanedioic acid), which is isomeric to MMA, also forms its diPFB ester. For the generation of mass spectra each of 17 µmol MMA and SA dissolved in 2 mL of acetonitrile were treated with 48 µmol PFB-Br in 200 µL of acetonitrile and 150 µmol of triethylamine and the mixtures were incubated at 65 °C for 2 h [6]. Interestingly, the diPFB esters of MMA and SA showed different mass and tandem mass spectra in LC-MS/MS in atmospheric pressure chemical ionization (APCI) mode. This is likely to be due to the C-H-acidity of MMA, which seems to play a role in APCI as well. Unfortunately, the retention times of the MMA and SA derivatives have not been reported in that article [6]. In serum (50 µL), MMA was converted to its diPFB ester by extractive derivatization with PFB-Br using tetrabutylhydrogen sulfate as the anion pair reagent [6]. 

The longer retention times, the larger mass fragments, and the smaller, later eluting GC-MS peaks suggest that these peaks are likely to contain three PFB residues. The most likely structures for these derivatives are 1,3-diPFB esters with an additional PFB moiety. Because the formation of these derivatives requires the presence of the Hünig base, it is reasonable to assume that the α-H atoms at C2 behave like acids and are substituted by PFB. Malondialdehyde is a C-H-acidic 1,3-dicarbonyl species and is converted to its 2,2-diPFB under considerably milder conditions in the absence of a base [5]. A triPFB derivative of MMA has not been reported thus far [6]. 

### 3.2. Quantitative Determination of MMA in Human Urine and Plasma by GC-MS

For the quantitative determination of MMA in human urine and plasma, we validated the GC-MS method using pooled human urine and plasma samples donated by a healthy volunteer. Urine (0, 40, 80, 120, 160, 200 µM) and plasma (0, 2, 4, 6, 8, 10 µM) samples were spiked with d_0_-MMA to reach relevant final concentrations. d_3_-MMA was used as the internal standard at a fixed added concentration of 100 µM for urine and 1 µM for plasma. All quantitative analyses were performed using oven program #2. To reach higher specificity, we chose the triPFB derivatives, which are not formed from the isomeric more abundant SA, and performed SIM of *m/z* 349 for d_0_-MMA and *m/z* 352 for the internal standard d_3_-MMA.

### 3.3. Method Validation in Human Urine and Plasma

The method was partially validated as described for endogenous substances [15], which are ubiquitous in varying concentrations in human biological samples including urine and plasma. The validation included standardization of the internal standard, linearity, precision (expressed as the coefficient of variation, CV), accuracy (expressed as the recovery rate) and limits of detection and quantitation. The stability of MMA in human plasma and urine was not investigated, because it is well known. The stability of the MMA derivatives in toluene extracts was not investigated, because such derivatives of several analytes are known to be stable for several weeks [14].

The peak area ratio (PAR) of *m/z* 349 for d_0_-MMA to *m/z* 352 for d_3_-MMA from analyses of equimolecular (100 µM) aqueous solutions of d_0_-MMA and d_3_-MMA was determined to be 0.920 ± 0.048 (CV, 5 %). The PAR differs by 8 % from the nominal molar ratio of 1:1.

Linear relationships were obtained between the PAR measured (*y*) and the concentration of d_0_-MMA (*x*) added to the urine (range, 0–200 µM) and plasma (range, 0–10 µM) samples using oven temperature program #2. These concentration ranges cover concentrations of endogenous MMA reported in the literature for healthy and ill subjects. The analyses were performed in duplicate for each concentration point and both matrices. The regression equations were: *y* = 0.112 + 0.0103 *x*, *r*^2^ = 0.9963, for urine; *y* = 0.20 + 0.823 *x*, *r*^2^ = 0.9796, for plasma. The coefficient of variation (CV) ranged between 1 and 14 % in urine, and between 0 and 19 % in plasma indicating acceptable precision of the method for these matrices. The slope values of the regression equations correspond to the mean recovery rate (accuracy) of the method for urine and plasma which is determined to be 103% and 82.3%, respectively. The *y*-axis intercepts indicate endogenous MMA at a mean concentration of 112 µM in the urine samples and 2.0 µM in the plasma samples used in method validation. In the validation experiments, the mean peak area of the internal standard d_3_-MMA was 2.0 × 10^7^ arbitrary units (CV, 9.4%) in the urine samples (100 µM) and 2.9 × 10^5^ arbitrary units (CV, 16.8%) in the plasma samples (1 µM), indicating similar extraction rates from urine and plasma samples.

The lowest d_0_-MMA concentration of 40 µM added to the validation urine sample was determined with a precision of 2.2% and an accuracy (recovery rate) of 90.3%. The value of 40 µM is considered the lower limit of quantitation (LLOQ) for this human urine. Under consideration of the basal MMA concentration of 112 µM, the relative LLOQ value is estimated to be 36%. This means that the method is able to accurately and precisely measure MMA at concentrations about 36% higher than the basal MMA concentration in human urine [15].

Linearity was also observed for the urine validation samples using oven temperature program #2. The retention time was 12.24 (0.03%) min for the d_3_-MMA derivative and 12.26 (0.03%) min for the d_0_-MMA derivative. The mean recovery rate was 87% and the precision (CV) was below 16% for d_0_-MMA added to urine (0–200 µM).

### 3.4. Measurement of MMA in Urine Samples of the Boys of the ASOS Study

**Figure 2:**
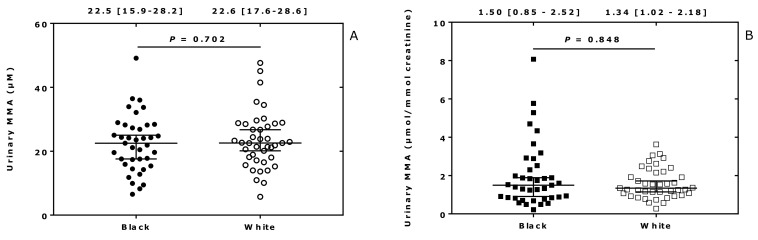
Methylmalonic acid (MMA) concentration (**A**) and creatinine-corrected excretion of MMA (**B**) in the urine of the black (*n* = 39) and white (*n* = 41) boys of the ASOS study.

The MMA concentration in the urine samples of all boys was determined to be 22.6 [17.2–28.2] µM. The creatinine-corrected excretion rate was determined to be 1.39 [0.91–2.28] µmol/mmol in all boys. The MMA concentration did not differ between black and white boys: 22.5 [15.9–28.2] µM vs. 22.6 [17.6–28.6] µM (*P* = 0.70; Mann–Whitney) (Figure 2A). The creatinine-corrected MMA excretion did not differ between black and white boys: 1.50 [0.85–2.52] µmol/mmol vs. 1.34 [1.02–2.18] µmol/mmol (*P* = 0.85; Mann-Whitney) (Figure 2B). The area under the curve (AUC) of the receiver operating characteristic (ROC) was 0.525 ± 0.065 (*P* = 0.697) for MMA in µM and 0.513 ± 0.067 (*P* = 0.844) for creatinine-corrected MMA. The MMA concentration (µM) in the urine samples of all boys was found to correlate after Spearman inversely with the boys’ age (*r* = -0.2394, *P* = 0.0324).

**Figure 3:**
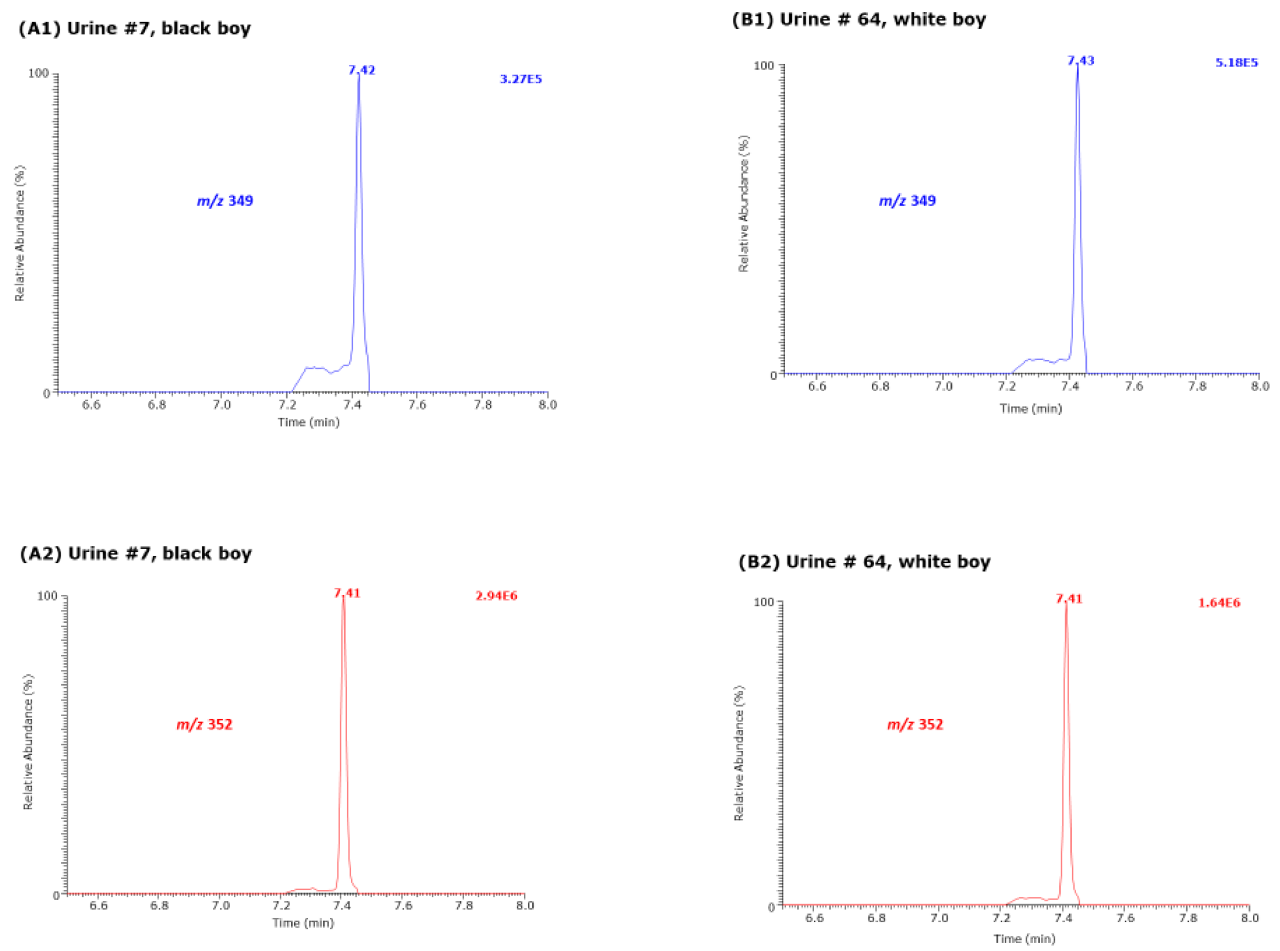
GC-MS chromatograms from quantitative analyses of methylmalonic acid (MMA) in urine samples (each 10 µL) of two boys of the ASOS study after derivatization with pentafluorobenzyl bromide. Selected-ion monitoring of *m/z* 349 for endogenous MMA (d_0_-MMA) (**A1**,**B1**) and of *m/z* 352 for the internal standard (d_3_-MMA) (**A2**, **B2**) was performed. The concentration of the internal standard was 100 µM in each urine sample. The oven program #2 was used for both urine samples. The signal-to-noise ratio values for d_3_-MMA (retention time, 7.41 min; *m/z* 352) were calculated as 58,905 in (**A2**) and 49,231 in (**B2**).

Exemplary GC-MS chromatograms from quantitative analyses of MMA in urine samples of two boys in the ASOS study are shown in Figure 3. Endogenous MMA and its standard eluted each as single peaks. The derivative of the internal standard d3-MMA eluted in front of the endogenous MMA due to the weaker interaction of the three deuterium atoms of the methyl group of the derivatives with the stationary phase of the column. In the quantitative analyses of MMA in the ASOS urine samples, the retention times were 7.41 ± 0.003 (CV, 0.04 %) min for the d_3_-MMA derivative and 7.425 ± 0.005 (CV, 0.07 %) min for the d_3_-MMA derivative. In these analyses, the peak area values varied by 24 % for the d_0_-MMA derivative and by 36 % for the d_3_-MMA derivative.

The GC-MS peak of the triPFB derivative of d_3_-MMA was used to estimate the lowest limit of detection (LLOD) of the method using a signal-to-noise (*S/N*) ratio of 3:1. The LLOD of the method was determined. Injection of 5 pmol of the triPFB derivative of d_3_-MMA produced peaks with *S/N* of 49,231 and 58,905 (s. 3). Approximating these values to an *S/N* ratio of 3:1 yields LLOD values of 255 amol d_3_-MMA and 305 amol d_3_-MMA, respectively. The LLOD values would translate to MMA concentrations of the order of 5 nM in human urine.

### 3.5. Methods Comparison for MMA

MMA has been analyzed in human serum and urine by GC with flame ionization detection (FID) after derivatization with BSTFA [16]. Reported MMA concentrations were 21.4 mg/L (181 µM) in the urine of healthy subjects and 478 mg/L (4051 µM) in the urine of a patient with methylmalonic aciduria [16]. MMA has been also analyzed by GC-FID after derivatization with BF_3_-propanol [17]. MMA was measured in the serum of healthy humans by GC-MS after derivatization with *N*-methyl-*N*(*t*-butyldimethylsilyl)trifluoroacetamide (MTBSTFA). Reported MMA concentrations ranges between 64 nM to 331 nM [18]. MMA and other carboxylic acids including amino acids were measured by GC-MS after derivatization with methylchloroformate [19]. Median plasma and serum MMA concentrations were reported to be 160 to 180 nM. The MMA concentration in human serum samples was measured by LC-MS/MS after derivatization with PFB-Br and found to range between 100 and 800 nM as measured [6]. More recently, MMA was measured in human urine as a marker of B_12_ deficiency by LC-MS/MS [20]. The creatinine-corrected excretion rate of MMA measured by this method ranged between 0.42 and 39.9 µmol/mmol creatinine (1.22 [0.80–1.81] µmol/mmol) [20]. Measurement by GC-MS upon derivatization with BSTFA (30 min, 70 °C) revealed similar results (range, 0.53 to 42.5 µmol/mmol (1.23 [0.90–2.08] µmol/mmol). The MMA excretion rates measured in the urine samples of the boys in the ASOS study by the present GC-MS method are very close to those measured by LC-MS/MS and GC-MS [20]. Elevated MMA concentrations are sensitive indicators of vitamin B_12_ deficiency, which is a worldwide problem, including selected areas in South Africa [21]. Our results suggest that the boys involved in the ASOS study do not suffer from vitamin B_12_ deficiency. 

## 4. Conclusions

MMA (CH_3_CH(COOH)_2_) is a very short dicarboxylic acid. The carboxylic groups of MMA are susceptible to derivatization with PFB-Br in aqueous acetone. Our study suggests that the C-2 H atom of MMA is C-H-acidic and can be substituted by a PFB residue from PFB-Br. At 80 °C and in the presence of *N,N*-diisopropylamine as the catalyst MMA reacts with PFB-Br to form a tripentafluorobenzyl derivative (CH_3_CPFB(COOPFB)_2_). This derivatization is highly characteristic of MMA. Succinic acid, the isomer of MMA does not undergo such derivatization due to the lack of C-H acidic H atoms in the methylene groups. Commercially available d_3_-MMA (CD_3_CH(COOH)_2_) is useful as an internal standard for the quantitative analysis of MMA in human urine and plasma by GC-MS. d_3_-MMA concentrations as low as 17 nM in human urine should be reliably quantifiable. black and white boys from South Africa excrete closely comparable amounts of MMA in their urine. This finding suggests that in the bi-ethnic population there is no ethnic-related vitamin B_12_ deficiency.

## Data Availability

Not available.

## Sample Availability

Not available.
